# Comprehensive Genomic Profiling of Small-Cell Lung Cancer Reveals Frequent Potentially Targetable Alterations

**DOI:** 10.3390/ijms262311512

**Published:** 2025-11-27

**Authors:** Dániel Schmalz, Zoltán Krabóth, Veronika Czoma, Péter Urbán, Attila Gyenesei, István Ruzsics, Veronika Sárosi, Árpád Boronkai, Emőke Papp, Béla Kajtár

**Affiliations:** 1 Center of Omics, János Szentágothai Research Center, University of Pécs, Ifjúság Street 20, 7624 Pécs, Hungary; schmalz.daniel@pte.hu (D.S.); urban.peter@pte.hu (P.U.); gyenesei.attila@pte.hu (A.G.); 2 Department of Pathology, Clinical Centre, University of Pécs, Szigeti Street 12, 7624 Pécs, Hungary; kraboth.zoltan@pte.hu (Z.K.); czoma.veronika@pte.hu (V.C.); 3 1st Department of Internal Medicine, Clinical Centre, University of Pécs, Ifjúság Street 13, 7624 Pécs, Hungary; ruzsics.istvan@pte.hu (I.R.); sarosi.veronika@pte.hu (V.S.); 4 Department of Oncotherapy, Clinical Centre, University of Pécs, Édesanyák Street 17, 7624 Pécs, Hungarypapp.emoke@pte.hu (E.P.)

**Keywords:** SCLC, comprehensive genomic profiling, NGS, copy number changes, *TYRO3*

## Abstract

Small-cell lung carcinoma (SCLC) remains one of the most aggressive lung cancers and continues to pose a major challenge for precision oncology. Despite its morphological uniformity, SCLC exhibits marked molecular heterogeneity with recurrent, potentially targetable genomic alterations. Comprehensive profiling is often hindered by limited tissue availability and the need for rapid therapeutic intervention. We performed genomic profiling of 55 primary and metastatic SCLC samples using a 324-gene hybrid-capture next-generation sequencing panel. Consistent with prior reports, nearly all tumors exhibited biallelic *TP53* and *RB1* inactivation. Recurrent alterations involved the PI3K/Akt/mTOR pathway (62%), chromatin regulators (42%), and NOTCH signaling genes (15%). *PTEN* mutations were enriched in brain metastases. Frequent copy-number gains affected *SOX2*, *NKX2-1*, MYC-family genes, and *CCNE1*. Two novel recurrent amplifications of potential clinical significance were identified: *TYRO3* (33%) and *SDHA* (13%). *TYRO3*, a TAM family receptor tyrosine kinase, and *SDHA*, a mitochondrial enzyme involved in succinate metabolism, may contribute to tumor progression and represent emerging therapeutic vulnerabilities. These findings underscore the genomic diversity of SCLC and highlight the potential utility of broad next-generation sequencing in uncovering new molecular targets for precision therapy.

## 1. Introduction

Small-cell lung carcinoma (SCLC) is the most aggressive form of lung cancer, accounting for approximately 15% of cases. It occurs predominantly in heavy smokers and is characterized by neuroendocrine differentiation, rapid proliferation, and early metastatic spread [1]. Although initially responsive to chemotherapy, relapse is almost inevitable, resulting in limited long-term survival. SCLC is clinically categorized as limited or extended stage disease, the former being confined to a single thoracic radiation field. The five-year overall survival for extended stage SCLC remains below 6% [2]. The addition of immune checkpoint inhibitors to the standard platinum-based chemotherapy has modestly improved survival in extended stage cases, but only a minority of patients benefit, largely due to the profoundly immunosuppressive tumor microenvironment [3].

Molecular subtypes of SCLC have been delineated based on transcriptional profiles, each associated with distinct biological features. The most common is the ASCL1-defined SCLC-A subtype, representing 40–50% of cases and characterized by high neuroendocrine marker expression. The NEUROD1-driven subtype (SCLC-N) typically shows lower neuroendocrine marker expression, whereas the POU2F3-defined subset (SCLC-P) is largely without classical neuroendocrine markers. A fourth subtype was originally proposed to be defined by YAP1 expression (SCLC-Y); however, more recent studies suggest that the fourth lineage is better represented by an inflammation-associated state (SCLC-I) [4]. Despite their biological relevance, these classifications do not yield actionable targets or predictive biomarkers, limiting their clinical utility [3]. Genomic studies remain constrained by frequent necrosis and the scarcity of available tumor tissue. Although analysis of cell-free DNA and circulating tumor cells offers a promising alternative, its clinical applicability is still being defined [5].

Previous genomic studies have typically examined a relatively low number of patients with few investigating more than 50 SCLC cases, often enriched for limited-stage disease amenable to surgical resection [2,6,7,8,9,10,11]. A recent large-scale study analyzing 3600 SCLC tumor samples, primarily of European ancestry, provided valuable insights regarding incidence of genomic alterations and associations with clinical features [5]. There is, however, a wide variability in mutation frequencies across cohorts and methodologies.

The present study reports real-world comprehensive genomic profiling of 55 Hungarian limited and extended stage SCLC patients, including both primary tumors and metastases. Using a 324-gene hybrid-capture panel applicable to formalin-fixed paraffin-embedded and cytological specimens, we characterized the landscape of genetic alterations and evaluated their associations with clinicopathological features.

## 2. Results

### 2.1. Recurrent Small Genetic Variants

**Figure 1:**
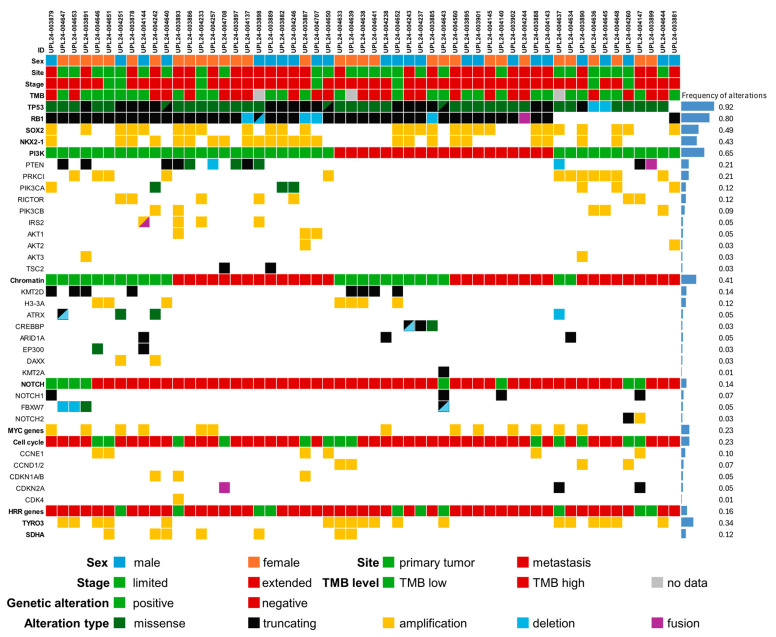
Landscape of genomic alterations found in the cohort studied.

*TP53* inactivation was detected in 54 of 55 samples (98%), appearing biallelic in all cases based on the presence of two independent mutations, homozygous deletion, or a single mutation with a variant allele frequency (VAF) indicative of loss of the wild-type allele. Twenty tumors harbored truncating *TP53* mutations, including five splice-site and 15 frameshift variants, while 35 carried missense mutations (Figure 1, Supplementary Table S1).

*RB1* inactivation was identified in 45 samples (82%). Homozygous deletions were detected in 4 cases, combined deletion and mutation in 1, and VAFs suggesting loss of wild-type *RB1* in 40 cases. One sample harbored two distinct heterozygous *RB1* mutations.

Alterations activating the PI3K/Akt/mTOR signaling pathway were present in 34 of 55 cases (62%). *PTEN* inactivation occurred in 22%, activating *PIK3CA* mutations in 5%, and inactivating *TSC2* mutations in 4%. Amplifications of *PRKCI*, *RICTOR*, *PIK3CA*, *PIK3CB*, *AKT1*, or *IRS2* were identified in an additional 34% of cases. Notably, all tumors lacking *RB1* alterations showed genetic activation of the PI3K/Akt/mTOR pathway (*p* = 0.002, Supplementary Table S3).

Mutations affecting chromatin regulators—including histone methyltransferases, acetyltransferases, and genes modulating chromatin accessibility—were observed in 42% of cases. The most frequently altered genes were *KMT2D* (15%), *CREBBP* (7%), *ARID1A* (5%), and *ATRX* (5%), followed by *EP300* and *ASXL1* (two cases each). These alterations were mutually exclusive. Amplification of *H3F3A*, encoding the histone variant H3.3, was found in 13% of cases, often co-occurring with mutations in the above genes. *TET2*, encoding a DNA dioxygenase involved in epigenetic regulation, showed loss-of-function mutations in 5%.

Genes of the NOTCH signaling pathway were affected in 15% of cases (8/55), predominantly through loss-of-function mutations in *NOTCH1* (7%), followed by *FBXW7* inactivation (5%) and *NOTCH2* mutation (one case). *FBXW7* loss is expected to increase *NOTCH1* activity, and that of other oncogenic targets (e.g., *MYC*, *CCNE1*), and may influence chromatin architecture through impaired degradation of multiple regulatory proteins.

Defects in homologous recombination repair (HRR) genes were identified in 10 of 55 cases (18%), involving *ATR* (two cases), and *ATM*, *BRCA2*, *CDK12*, *NBN*, *FANCG*, *MLH1*, *RAD54L*, and *PARP2* (one case each). These alterations comprised truncating mutations or homozygous deletions and were significantly associated with *PTEN* loss (42% with mutation vs. 12% in PTEN-wild-type cases, *p* = 0.03).

The tumor mutation burden (TMB) was high across the cohort, with a mean of 12.7 mutations per megabase. Three samples could not be assessed with high confidence. Using a cutoff of 10 mutations/MB, 28 tumors exhibited high TMB, and 24 showed low TMB. All cases were microsatellite stable.

### 2.2. Recurrent Copy Number Changes

*SOX2* amplification was detected in 27 of 55 tumors (49%) and was more frequent in metastases (64%) than in primary tumors (30%) (*p* = 0.01). It was also associated with high tumor mutation burden (TMB) (64% in high vs. 33% in low TMB cases; *p* = 0.025).

*NKX2-1* amplification occurred in 24 of 55 tumors (44%), significantly enriched in metastases (81%) compared with primary tumors (19%) (*p* < 0.001). *SOX2* and *NKX2-1* were frequently coamplified (75% of *NKX2-1*- and 67% of *SOX2*-amplified cases showed amplification of the other gene, respectively; *p* < 0.001). Amplification of either gene correlated with the type of *TP53* mutation: truncating *TP53* variants predominated in *SOX2/NKX2-1*-amplified tumors (58% with vs. 19% without amplification; *p* = 0.003), whereas missense *TP53* mutations were more common in non-amplified cases (81% vs. 42%; *p* = 0.003).

*TYRO3*, encoding a receptor tyrosine kinase, was amplified in 18 cases (33%). *TYRO3* amplification correlated with mutations of chromatin regulators (57% with vs. 16% without amplification; *p* = 0.002) and was inversely associated with *SOX2* or *NKX2-1* amplification (11% with vs. 54% without *SOX2* amplification; *p* < 0.001).

Amplifications of *MYC* (5%), *MYCL* (13%), and *MYCN* (5%) were recurrent but mutually exclusive. MYC-family amplifications were associated with *SOX2* amplification (37% with vs. 11% without; *p* = 0.023) and were more frequent in tumors lacking *TYRO3* amplification (6% with vs. 32% without *TYRO3* amplification; *p* = 0.025).

Genetic alterations promoting cell cycle progression were identified in 13 of 55 cases (24%), including *CCNE1* amplification (11%), *CCND1* amplification (7%), *CDK4* amplification (one case), and *CDKN2A* inactivation (one case).

*SDHA* amplification was detected in seven tumors (13%). *TET2* or *ASXL1* mutations were significantly associated with *SDHA* amplification (43% with vs. 4% without; *p* = 0.012).

Amplification of any gene located within the 3q chromosomal region was observed in 37 of 55 cases (67%), including *KLHL6*, *SOX2*, *PRKCI*, *PIK3CA*, *PIK3CB*, and *TERC*. *KLHL6*, *PRKCI*, and *TERC* were frequently coamplified, whereas *SOX2* and *PRKCI* amplifications tended to be mutually exclusive: *SOX2* amplification occurred in 25% of *PRKCI*-amplified tumors versus 56% of PRKCI-wild-type cases (*p* = 0.058; not reaching statistical significance).

Finally, six tumors (11%) showed amplification of *BCL2*, *BCL2L1*, or *BCL2L2*, consistent with enhanced apoptotic resistance and increased cell survival.

### 2.3. Genetic Alterations Recurrent in NSCLC

Genetic alterations more typically associated with non-small-cell lung carcinoma (NSCLC) were identified in seven cases. One patient—a never-smoker male—harbored an *EGFR p.(L858R)* activating mutation detected in a liver metastasis. He had been diagnosed with lung *EGFR*-mutated adenocarcinoma 28 months earlier. Following initial treatment with gefitinib, disease progression occurred after 14 months, at which time an *EGFR p.(T790M)* resistance mutation was identified by liquid biopsy. The patient was subsequently treated with osimertinib, achieving stable disease for 14 months, after which multiple liver metastases developed. Liver biopsy at that time revealed histologic transformation to SCLC. The transformed tumor retained the same *EGFR p.(L858R)*, and *RB1*, *TP53*, and *PIK3CA* mutations as the original adenocarcinoma, while showing RICTOR amplification as a novel change.

In five additional cases, tumors carried *KRAS* or *HRAS* activating mutations or *BRAF* fusions, with no clinical evidence of a preceding NSCLC diagnosis. A further SCLC tumor exhibited a *SMARCA4* loss-of-function mutation. Although histologic evaluation was limited by crush artifact, cytomorphologic and immunophenotypic features were consistent with SCLC, showing TTF1 expression and no consistent loss of SMARCA4 positivity by immunohistochemistry.

### 2.4. Correlation with Clinical Parameters

*PTEN* mutations and MYC-family amplifications (*MYC*, *MYCL*, or *MYCN*) were more frequent in brain metastases (43% and 38%, respectively) than in other sites (9% and 15%, respectively; *p* = 0.004 and *p* = 0.05, Table 1). Primary tumors more often harbored alterations in epigenetic regulators than metastatic samples (58% primary vs. 24% metastatic tumor; *p* = 0.012). *TYRO3* amplification showed a similar pattern, being more frequent in primary tumors (65% primary vs. 3% metastatic tumor; *p* < 0.001). *NKX2-1* and *SOX2* amplifications were more common in metastases (*p* < 0.001 and *p* = 0.010).

Female patients exhibited a higher frequency of *PTEN* mutations (32% in females vs. 8% in males; *p* = 0.033) and missense *TP53* mutations (81% vs. 42%; *p* = 0.003), whereas truncating *TP53* mutations were less common in females (19% in females vs. 58% in males; *p* = 0.003, Table 2).

No genomic alterations showed significant correlation with overall survival, and tumors from patients with exceptionally long survival displayed alteration frequencies comparable to those of other cases (Supplementary Table S2).

## 3. Discussion

SCLC remains the most aggressive and lethal malignancy of the lung, with limited advances in targeted therapy and predictive biomarker development. Although immunotherapy has provided a modest survival benefit, only a minority of patients derive durable responses. Molecular subtyping of SCLC based on transcriptional profiles has recently been established, yet the clinical relevance of these categories remains incompletely understood [12]. Comprehensive genomic profiling of SCLC is therefore essential to identify actionable alterations and improve patient stratification; however, tumor heterogeneity and the limited availability of adequate tissue have hindered such efforts.

In this study, we demonstrated the feasibility of high-depth, DNA-based comprehensive genomic profiling using a targeted 324-gene panel, which enabled concurrent detection of single-nucleotide variants, copy number alterations, and tumor mutation burden (TMB), even from cytology-derived samples.

Consistent with previous reports, *TP53* and *RB1* were the most frequently altered genes, showing biallelic inactivation in 98% and 82% of cases, respectively—values at the upper end of published ranges (*TP53*: 80–97%, *RB1*: 60–80%) [2,5,6,7,8,9,10,11]. Interestingly, missense mutations were more common in female patients (Table 2). This difference between female and male patients has not been reported before and may reflect a sex-based disparity of *TP53* gene expression and activity of gain-of-function mutations, as has been suggested previously related to tumorigenesis and cancer progression [13,14].

Alterations activating the PI3K/Akt/mTOR pathway were highly prevalent, affecting 62% of tumors. *PTEN* mutations occurred in 22% of cases and were strongly enriched in brain metastases (43% vs. 9% in other sites; *p* = 0.004), consistent with prior studies reporting a similar association [2,5,10]. This observation mirrors findings in NSCLC and other tumor types, where *PTEN* loss has been implicated in brain tropism [15,16] and aligns with the established role of *PTEN* in glioblastoma pathogenesis [17]. *PIK3CA* and *TSC2* mutations were observed in 5% and 4% of cases, respectively, comparable to published frequencies (2–10%) [2,5,10,11]. In addition, amplifications of *PRKCI*, *RICTOR*, *PIK3CA*, *PIK3CB*, *IRS2*, or *AKT1* were detected in 35% of cases, collectively indicating widespread pathway activation. *RICTOR* amplification occurred in 14% of cases, in line with previous reports, and it was suggested to be predictive of responsiveness to mTOR inhibition [18]. Although therapeutic blockade of PI3K/Akt/mTOR signaling remains a promising avenue, optimal patient selection and rational combination strategies are yet to be defined [19]. *PRKCI* amplification was detected in 22% of our cohort. *PRKCI*, encoding an atypical protein kinase C isoform, has been described as an oncogenic driver in ovarian and squamous lung carcinomas [20]. It contributes to tumorigenesis by disrupting cell polarity and suppressing autophagy and has been linked to poor prognosis in multiple cancer types [20]. The antirheumatic drug auranofin was recently shown to inhibit *PRKCI* and sensitize SCLC cells to sorafenib in vitro [21,22], highlighting a potential therapeutic avenue worth clinical exploration.

Amplifications of *MYC*, *MYCL*, and *MYCN* were found in 23% of cases, within the 6–25% range reported in the literature. MYC-family amplifications have been associated with increased sensitivity to aurora kinase inhibitors and poor prognosis, though no survival correlation was observed in our cohort [4,23,24].

Genetic alterations influencing epigenetic regulation are recurrent in SCLC. *KMT2D* (also known as *MLL2*) has been reported in 10–15% cases [5,6,7,10], *CREBBP* and *EP300* mutations are also reported with 2–18% incidence [2,5,25]. These genes encode histone methyltransferase or acetyltransferase enzymes; their loss may alter chromatin accessibility, gene expression, and immune recognition [26]. *ARID1A* encodes a subunit of SWI/SNF chromatin remodeling complex that also contributes to the above-mentioned functions regarding chromatin maintenance. *ARID1A* mutations are also recurrent, occurring in 3–5% of cases. Alterations of chromatin remodeling have been reported to potentially contribute to immune evasion of cancer cells by downregulating MHC-I molecules and components of antigen presentation machinery [26]. It has been proposed that loss-of-function mutations of these genes may lead to reduced neuroendocrine identity and increased plasticity in SCLC, promoting survival and resistance to therapy [26]. *ATRX* encodes a chromatin remodeling protein that interacts with H3.3 histone proteins and is mutated in 2% of SCLC cases [5]. *TET2* encodes a protein involved in DNA methylation affecting epigenetic regulation and is recurrently mutated in 1–2% of SCLC cases [5]. Our cohort showed mutations in the above-mentioned genes involved in epigenetic regulation in 42% of cases. Preclinical data suggest that patients harboring these alterations might benefit from BET, EZH2, or HDAC inhibitors, particularly in combination with immune checkpoint blockade [26].

Amplification of *H3F3A*, encoding histone variant H3.3, was observed in 13% of cases. *H3F3A* amplification or overexpression has been linked to enhanced migration and aggressive behavior in lung and esophageal cancers [27]. Activating mutations of *H3F3A* have been reported in gliomas and giant cell tumors of bone. Amplification has been described as recurrent in approximately 7.5% of esophageal carcinoma and was seen to be associated with shortened survival [28]. Similarly, *SDHA* amplification was detected in 13% of tumors and correlated with *TET2* or *ASXL1* mutations. *SDHA* encodes a mitochondrial enzyme; its amplification may alter cellular metabolism through changes in succinate and α-ketoglutarate levels, potentially influencing the activity of epigenetic regulators such as *TET2* and *ASXL1*, and immune modulation within the tumor microenvironment. *SDHA* amplification has been described in ovarian and breast carcinomas, where it promotes metabolic reprogramming and sensitivity to agents such as shikonin [29,30]. Given preclinical reports that shikonin suppresses SCLC cell growth [31], *SDHA* amplification may represent a novel predictive biomarker warranting further investigation.

*TYRO3* amplification was found in 33% of tumors, predominantly in primary sites. *TYRO3*, part of the TAM receptor tyrosine kinase family along with *AXL* and *MERTK*, regulates local anticoagulation, proliferation, migration, and immune evasion [32,33]. Overexpression of *TYRO3* has been associated with resistance to immune checkpoint blockade via inhibition of ferroptosis [34]. Increased Dtk (a synonym of Tyro3) expression has been described in SCLC cell lines [35]. Although the TAM inhibitor sitravatinib failed to improve outcomes in unselected NSCLC patients when added to immunotherapy [36], *TYRO3* amplification may identify a subset of SCLC patients who could benefit from TAM-targeted therapies [37].

Defects in homologous recombination repair (HRR) genes were observed in 18% of tumors, a frequency comparable to recent studies [38]. HRR-deficient tumors often exhibit sensitivity to platinum compounds and PARP inhibitors. In our cohort, HRR gene alterations were not associated with *KMT2D* mutation or high TMB as reported previously [38], but correlated with *PTEN* loss, suggesting a potential link between genomic instability and PI3K pathway deregulation. Studies have described germline pathogenic mutations of HRR genes like *BRCA2*, *CHEK1* and *RAD51D* in SCLC patients [39] and have investigated the potential benefit of PARP inhibitors in SCLC therapy [40].

The most frequent alteration overall was *SOX2* amplification (49%), which correlated with high TMB, *NKX2-1* amplification, and metastatic localization. *SOX2* plays a key role in neuroendocrine differentiation and pluripotency and is reported in 20–30% of SCLC cases [1]. Although *NKX2-1* amplification is rarely described (2–3%), it is often expressed and is characteristic of the SCLC-A molecular subtype [41].

SCLC may arise from transformation of NSCLC, especially *EGFR*-mutated adenocarcinoma. In 13% of cases, we identified genomic alterations more characteristic of NSCLC, including *EGFR*, *KRAS*, and *HRAS* activating mutations, *BRAF* fusions, and *SMARCA4* loss-of-function mutations. Except for a single case of histologically confirmed adenocarcinoma transformation, there was no clinical evidence of prior NSCLC. *SMARCA4* mutations have been described in a small subset of SCLC but may also reflect SMARCA4-deficient undifferentiated carcinomas, a recently defined entity [5,42].

None of the genomic alterations identified in our study showed significant association with overall survival. Although amplification of 4q12 (including *KIT*, *KDR*, and *PDGFRA*) was recently linked to improved outcomes [5], no survival advantage was observed in our 4q12-amplified cases (5%), most likely attributable to the limited cohort size.

In summary, our comprehensive genomic analysis of 55 SCLC tumors revealed recurrent, potentially targetable alterations in the PI3K/Akt/mTOR, NOTCH, and chromatin remodeling pathways, and in genes involved in homologous recombination repair. Importantly, we report *TYRO3* amplification for the first time in SCLC, detected in one-third of cases, suggesting a novel mechanism of tumor progression and a potential therapeutic vulnerability. It is important to note that published SCLC datasets—including the recent large real-world series by Sivakumar et al. [5]—exhibit substantial variability in mutation rates across genes and pathways. These differences may reflect heterogeneity in patient populations, sample types, sequencing platforms, and analytical pipelines rather than true biological divergence. Accordingly, our findings should be viewed as complementary to the larger datasets, while acknowledging that geographic or cohort-specific effects cannot be excluded. Validation in larger, multi-ethnic patient cohorts and functional studies is warranted to confirm the biological and clinical relevance of *TYRO3* amplification in SCLC.

## 4. Materials and Methods

### 4.1. Patients and Samples

A total of 55 samples with adequate DNA of patients diagnosed with small-cell lung carcinoma (SCLC) at the Pathology Department of University of Pécs were included in this retrospective study. The mean age at diagnosis was 64 years (range, 43–87 years); 31 patients were female, and 24 were male (Table 3, Supplementary Table S4). All but one patient had a history of smoking.

Twenty-nine tumor samples were obtained from metastatic sites, most commonly from the central nervous system (*n* = 21), followed by the liver (*n* = 4), gastrointestinal tract (*n* = 2), lymph node (*n* = 1), and dermis (*n* = 1). The remaining 26 samples were derived from primary pulmonary tumors.

Cytological smears were available for 21 cases, while formalin-fixed, paraffin-embedded (FFPE) tissue blocks were used in 34. Tumor cellularity exceeded 20% in all specimens, with a mean tumor content of 75%.

Most patients received platinum-based chemotherapy. Ten patients underwent no systemic therapy or received palliative radiotherapy only, while treatment data were unavailable for 10 patients. The median follow-up duration was 8.5 months. Ten patients exhibited prolonged survival (>24 months), and survival data were not available for seven patients.

### 4.2. Comprehensive Genomic Profiling

Following pathological review and assessment of tumor purity, DNA was extracted using an automated purification system (Ion Torrent Genexus Purification System; FFPE DNA and RNA Purification Kit, Thermo Fisher Scientific, Waltham, MA, USA) according to the manufacturer’s instructions.

Comprehensive genomic profiling was performed using the AVENIO Tumor Tissue Comprehensive Genomic Profiling (CGP) Kit (Roche, Switzerland), a research-use-only, DNA-based hybrid-capture next-generation sequencing (NGS) assay targeting 324 cancer-associated genes (Supplementary Table S5). The panel enables detection of single-nucleotide variants (SNVs), insertions/deletions (indels), copy number variations (CNVs), and structural rearrangements, and estimation of tumor mutational burden (TMB) and microsatellite instability (MSI). The AVENIO CGP gene content and secondary analysis pipeline are aligned with the clinically validated FoundationOne CDx (F1CDx) FoundationOne (Cambridge, MA, USA) platform, which is FDA-approved as an in vitro diagnostic assay.

Sequencing was performed on an Illumina NextSeq 550 instrument (Illumina, San Diego, CA, USA) using eight indexed libraries per run. Primary and secondary data processing were conducted with the FoundationOne Analysis Platform, applying algorithms previously described by Milbury et al. [43]. Tertiary analysis was performed using Navify Mutation Profiler (version 2.4.1; Roche, Switzerland).

Variant calling thresholds were defined as ≥4% variant allele frequency (VAF) for SNVs/indels, ≥6 copies for amplifications, and ≥3 supporting reads for fusions. Oncogenic potential of small variants was further assessed using VarSeq (version 2.6.2; Golden Helix, USA) and refined by manual curation. Truncating variants in known oncogenes, missense variants with low predicted pathogenicity (REVEL score < 0.7), no functional data and low allele frequency in COSMIC (<15 counts), and fusions lacking critical functional domains of genes involved were classified as variants of uncertain significance (VUS) with minimal likelihood of oncogenicity and were excluded from downstream analyses.

### 4.3. Statistical Analysis

SPSS 30.0 software (SPSS Inc., Chicago, IL, USA) was used for statistical analysis. The significance of different incidence values was determined by the χ^2^ test or Fisher’s exact test, as appropriate. Associations with a *p*-value < 0.05 were considered statistically significant.

## 5. Conclusions

SCLC is an aggressive cancer type with a dismal prognosis and significant genetic heterogeneity. Genomic profiling may provide predictive information for novel targeted therapeutic approaches. Rapid comprehensive genomic profiling is possible in SCLC even when only cytological samples are available. Further clinical research may lead to the much-needed improvement of outcomes for patients with SCLC.

## Figures and Tables

**Table 1:** Genomic alterations at primary and metastatic sites.

Genomic Alteration (N, %)	Primary Tumors (*n* = 26)	Metastatic Sites (*n* = 29)	Significance (*p* Value)
*TP53* gene	26 (100%)	28 (97%)	0.527
missense	19 (73%)	16 (55%)	0.136
truncating	8 (31%)	12 (41%)	0.297
*RB1* gene	19 (73%)	26 (90%)	0.107
PI3K/Akt/mTOR pathway	17 (65%)	17 (59%)	0.407
PTEN *	3 (12%)	9 (31%)	0.076
Chromatin regulators	16 (62%)	7 (24%)	0.005
NOTCH pathway	5 (19%)	3 (10%)	0.291
MYC-family *	5 (19%)	8 (28%)	0.342
Cell cycle genes	7 (27%)	6 (21%)	0.410
Homologous repair genes	5 (19%)	5 (17%)	0.561
*SOX2*	8 (31%)	19 (66%)	0.010
*NKX2-1*	5 (19%)	19 (65%)	<0.001
*TYRO3*	17 (65%)	1 (3%)	<0.001
*SDHA*	4 (15%)	3 (10%)	0.437

The table shows number and percentage of cases with genetic alterations involving genes or genetic pathways. * Metastatic sites in case of *PTEN* mutation and MYC-family amplification were exclusively the brain; *PTEN* mutation: 3/34 any site vs. 9/21 brain metastasis, *p* = 0.004; MYC-family amplification: 5/34 any site vs. 8/21 brain metastasis, *p* = 0.050).

**Table 2:** Genomic alterations and association with sex.

Genomic Alteration	Total (*n* = 55)	Female (*n* = 31)	Male (*n* = 24)	Significance (*p* Value)
*TP53* gene	54 (98%)	31 (100%)	23 (96%)	0.436
missense	35 (64%)	25 (81%)	10 (42%)	0.003
truncating	20 (36%)	6 (19%)	14 (58%)	0.003
*RB1*	45 (82%)	25 (81%)	20 (83%)	0.542
PI3K/Akt/mTOR pathway	34 (62%)	20 (65%)	14 (58%)	0.424
*PTEN*	12 (22%)	10 (32%)	2 (8%)	0.033
Chromatin regulators	23 (42%)	15 (48%)	8 (33%)	0.199
NOTCH pathway	8 (15%)	6 (19%)	2 (8%)	0.225
MYC-family	13 (24%)	5 (16%)	8 (33%)	0.121
Cell cycle genes	13 (24%)	9 (29%)	4 (17%)	0.228
Homologous repair genes	10 (18%)	5 (16%)	5 (21%)	0.458
*SOX2*	27 (49%)	12 (39%)	15 (63%)	0.069
*NKX2-1*	24 (44%)	11 (36%)	13 (54%)	0.133
*TYRO3*	18 (33%)	13 (42%)	5 (21%)	0.085
*SDHA*	7 (13%)	5 (16%)	2 (16%)	0.331

The table shows number and percentage of cases with genetic alterations involving genes or genetic pathways.

**Table 3:** Clinicopathologic characteristics of patients.

Characteristic	Total	Female	Male
Number of patients	55	31 (56%)	24 (44%)
Age, median (range)	64 (43–87)	65 (43–73)	64 (53–87)
Stage at diagnosis			
limited	7 (13%)	3 (10%)	4 (17%)
extended	48 (87%)	28 (90%)	20 (83%)
Survival, median	8.5	8.0	9.0
>24 months	10 (18%)	6 (19%)	4 (17%)
Site of biopsy			
primary tumor	26 (47%)	15 (48%)	11 (46%)
metastasis, brain	21 (38%)	12 (39%)	9 (38%)
metastasis, liver	4 (7%)	3 (10%)	1 (4%)
metastasis, other	4 (7%)	1 (3%)	3 (13%)
Biopsy type			
FFPE	34 (62%)	17 (55%)	17 (71%)
cytology	21 (38%)	14 (45%)	7 (29%)
Tumor purity (%)			
median (range)	81 (24–98)	80 (24–98)	84 (29–98)

The table shows number and percentage of cases with genetic alterations involving genes or genetic pathways.

## Data Availability

The datasets generated and/or analyzed in the present study are available from the corresponding author upon reasonable request.

## Supplementary Materials

The following supporting information can be downloaded at https://www.mdpi.com/article/10.3390/ijms262311512/s1.
