# Supplementary material for: Comprehensive Genomic Profiling of Small-Cell Lung Cancer Reveals Frequent Potentially Targetable Alterations
Source: Int J Mol Sci. 2025 Nov 27;26(23):11512. doi: 10.3390/ijms262311512 (PMC12692088; doi:10.3390/ijms262311512)
Supplement: Supplementary file 1 [file ijms-26-11512-s001.zip › Supplementary Table 5_revised.docx]

Supplementary Table 5. Gene list of AVENIO Tumor Tissue CGP Kit

| **Genes for SNV/indel and CNV (full coding regions)** |
| --- |
| ABL1, ACVR1B, AKT1, AKT2, AKT3, ALK, ALOX12B, AMER1(FAM123B), APC, AR, ARAF, ARFRP1, ARID1A, ASXL1, ATM, ATR, ATRX, AURKA, AURKB, AXIN1, AXL, BAP1, BARD1, BCL2, BCL2L1, BCL2L2, BCL6, BCOR, BCORL1, BRAF, BRCA1, BRCA2, BRD4, BRIP1, BTG1, BTG2, BTK, C11ORF30(EMSY), CALR, CARD11, CASP8, CBFB, CBL, CCND1, CCND2, CCND3, CCNE1, CD22, CD274(PD-L1), CD70, CD79A, CD79B, CDC73, CDH1, CDK12, CDK4, CDK6, CDK8, CDKN1A, CDKN1B, CDKN2A, CDKN2B, CDKN2C, CEBPA, CHEK1, CHEK2, CIC, CREBBP, CRKL, CSF1R, CSF3R, CTCF, CTNNA1, CTNNB1, CUL3, CUL4A, CXCR4, CYP17A1, DAXX, DDR1, DDR2, DIS3, DNMT3A, DOT1L, EED, EGFR, EP300, EPHA3, EPHB1, EPHB4, ERBB2, ERBB3, ERBB4, ERCC4, ERG, ERRFI1, ESR1, EZH2, FAM46C, FANCA, FANCC, FANCG, FANCL, FAS, FBXW7, FGF10, FGF12, FGF14, FGF19, FGF23, FGF3, FGF4, FGF6, FGFR1, FGFR2, FGFR3, FGFR4, FH, FLCN, FLT1, FLT3, FOXL2, FUBP1, GABRA6, GATA3, GATA4, GATA6, GID4(C17ORF39), GNA11, GNA13, GNAQ, GNAS, GRM3, GSK3B, H3F3A, HDAC1, HGF, HNF1A, HRAS, HSD3B1, ID3, IDH1, IDH2, IGF1R, IKBKE, IKZF1, INPP4B, IRF2, IRF4, IRS2, JAK1, JAK2, JAK3, JUN, KDM5A, KDM5C, KDM6A, KDR, KEAP1, KEL, KIT, KLHL6, KMT2A(MLL), KMT2D(MLL2), KRAS, LTK, LYN, MAF, MAP2K1(MEK1), MAP2K2(MEK2), MAP2K4, MAP3K1, MAP3K13, MAPK1, MCL1, MDM2, MDM4, MED12, MEF2B, MEN1, MERTK, MET, MITF, MKNK1, MLH1, MPL, MRE11A, MSH2, MSH3, MSH6, MST1R, MTAP, MTOR, MUTYH, MYC, MYCL(MYCL1), MYCN, MYD88, NBN, NF1, NF2, NFE2L2, NFKBIA, NKX2-1, NOTCH1, NOTCH2, NOTCH3, NPM1, NRAS, NT5C2, NTRK1, NTRK2, NTRK3, P2RY8, PALB2, PARK2, PARP1, PARP2, PARP3, PAX5, PBRM1, PDCD1(PD-1), PDCD1LG2(PD-L2), PDGFRA, PDGFRB, PDK1, PIK3C2B, PIK3C2G, PIK3CA, PIK3CB, PIK3R1, PIM1, PMS2, POLD1, POLE, PPARG, PPP2R1A, PPP2R2A, PRDM1, PRKAR1A, PRKCI, PTCH1, PTEN, PTPN11, PTPRO, QKI, RAC1, RAD21, RAD51, RAD51B, RAD51C, RAD51D, RAD52, RAD54L, RAF1, RARA, RB1, RBM10, REL, RET, RICTOR, RNF43, ROS1, RPTOR, SDHA, SDHB, SDHC, SDHD, SETD2, SF3B1, SGK1, SMAD2, SMAD4, SMARCA4, SMARCB1, SMO, SNCAIP, SOCS1, SOX2, SOX9, SPEN, SPOP, SRC, STAG2, STAT3, STK11, SUFU, SYK, TBX3, TEK, TET2, TGFBR2, TIPARP, TNFAIP3, TNFRSF14, TP53, TSC1, TSC2, TYRO3, U2AF1, VEGFA, VHL, WHSC1(MMSET), WHSC1L1, WT1, XPO1, XRCC2, ZNF217, ZNF703 |
| **Genes for select rearrangements** |
| ALK, BCL2, BCR, BRAF, BRCA1, BRCA2, CD74, EGFR, ETV4, ETV5, ETV6, EWSR1, EZR, FGFR1, FGFR2, FGFR3, KIT, KMT2A(MLL), MSH2, MYB, MYC, NOTCH2, NTRK1, NTRK2, NUTM1, PDGFRA, RAF1, RARA, RET, ROS1, RSPO2, SDC4, SLC34A2, TERC, TERT*, TMPRSS2 |

*TERT promoter region only
