# Supplementary material for: Comprehensive Genomic Profiling of Small-Cell Lung Cancer Reveals Frequent Potentially Targetable Alterations
Source: Int J Mol Sci. 2025 Nov 27;26(23):11512. doi: 10.3390/ijms262311512 (PMC12692088; doi:10.3390/ijms262311512)
Supplement: Supplementary file 1 [file ijms-26-11512-s001.zip › Supplementary Table 3.docx]

Supplementary Table 3. Associations between genetic alterations and survival

| **Genomic alteration** | **Extended survival**  **(> 24 months, n = 10)** | **Usual survival**  **(< 24 months, n = 38)** | **Significance**  **(p value)** |
| --- | --- | --- | --- |
| *TP53* gene  missense mutations  truncating mutations | 10 (100%)  7 (70%)  3 (30%) | 37 (97%)  25 (66%)  13 (34%) | 0.792  0.560  0.560 |
| *RB1* gene | 8 (80%) | 32 (84%) | 0.536 |
| PI3K/Akt/mTOR pathway  *PTEN* | 6 (60%)  3 (30%) | 23 (61%)  6 (16%) | 0.624  0.273 |
| Chromatin regulators | 5 (50%) | 15 (40%) | 0.401 |
| NOTCH pathway | 2 (20%) | 4 (11%) | 0.366 |
| MYC-family | 2 (20%) | 8 (21%) | 0.658 |
| Cell cycle genes | 4 (40%) | 7 (18%) | 0.153 |
| Homologous repair genes | 2 (20%) | 7 (18%) | 0.611 |
| *SOX2* | 4 (40%) | 18 (47%) | 0.479 |
| *NKX2-1* | 4 (40%) | 15 (40%) | 0.624 |
| *TYRO3* | 4 (40%) | 14 (37%) | 0.565 |
| *SDHA* | 2 (20%) | 4 (11%) | 0.366 |

The table shows number and percentage of cases with genetic alterations involving genes or genetic pathways.
