# Supplementary material for: Comprehensive Genomic Profiling of Small-Cell Lung Cancer Reveals Frequent Potentially Targetable Alterations
Source: Int J Mol Sci. 2025 Nov 27;26(23):11512. doi: 10.3390/ijms262311512 (PMC12692088; doi:10.3390/ijms262311512)
Supplement: Supplementary file 1 [file ijms-26-11512-s001.zip › Supplementary Table 2.docx]

Supplementary Table 2. Associations between genetic alterations and *TP53*/*RB1* positivity

| **Genomic alteration** | **TP53/RB1 inactivation**  **(n = 44)** | **no TP53/RB1 inactivation**  **(n = 11)** | **Significance**  **(p value)** |
| --- | --- | --- | --- |
| *TP53* gene  missense mutations  truncating mutations | 44 (100%)  28 (64%)  19 (43%) | 10 (91%)  7 (64%)  1 (9%) | 0.200  0.643  **0.036** |
| *RB1* gene | 44 (100%) | 1 (9%) | **<0.001** |
| PI3K/Akt/mTOR pathway  *PTEN** | 23 (52%)  9 (21%) | 11 (100%)  3 (27%) | **0.002**  0.449 |
| Chromatin regulators | 21 (48%) | 2 (18%) | 0.073 |
| NOTCH pathway | 6 (14%) | 2 (18%) | 0.508 |
| MYC-family* | 11 (25%) | 2 (18%) | 0.486 |
| Cell cycle genes | 9 (21%) | 4 (36%) | 0.232 |
| Homologous repair genes | 7 (16%) | 3 (27%) | 0.315 |
| *SOX2* | 22 (50%) | 5 (46%) | 0.527 |
| *NKX2-1* | 22 (50%) | 2 (18%) | 0.056 |
| *TYRO3* | 12 (27%) | 6 (55%) | 0.088 |
| *SDHA* | 7 (16%) | 0 (0%) | 0.189 |

The table shows number and percentage of cases with genetic alterations involving genes or genetic pathways.
